# Postgraduate students’ mental health, it is the time to be aware and act.

**DOI:** 10.1192/j.eurpsy.2024.712

**Published:** 2024-08-27

**Authors:** A. A. Alageel

**Affiliations:** Psychiatry Department, College of Medicine, Imam Mohammad Ibn Saud Islamic University (IMSIU), Riyadh, Saudi Arabia

## Abstract

**Introduction:**

Education is markedly associated with well‐being, leading to the acquisition of healthy behaviors while at the same increasing hiring and salary. However, stress among academics is worrying, especially in younger researchers who experience significant levels of job insecurity, the imbalances between life and job, stressful relationships with supervisors and funding difficulties. Several studies have indicated that most graduate students spent over 40 hours per week on their postgraduate program, more than 70 % were not able to complete their programs within the set timeframe, and had uncertainty related to their job

**Objectives:**

In this review, we discuss the mental health of postgraduate students focusing on depression, anxiety, stress, and smartphone addiction.

**Methods:**

a review presentation of the mental health of postgraduate

**Results:**

According to meta-analysis, depression prevalence among postgraduate participants ranges from 6.2% to 85.4% in 36 studies. The pooled prevalence was 34% (26,579 individuals; 95% CI: 28–40). A study using the GAD-7 scale to evaluate the prevalence of anxiety concluded that 41% of postgraduate students suffered moderate to severe GAD, which is about six times the prevalence of GAD among the general population. A Study demonstrated 51.0% of the participants had smartphone addiction. A significant association was also observed between extensive smartphone use and depression (*P* = 0.001). Of the smokers in this study, 41.5% were addicted to smartphones (*P* = 0.039). Smartphone addicts had approximately two times the chance of having insomnia (OR = 2.113) (*P* = 0.013). In addition, they showcased more ADHD symptoms (OR = 2.712) (*P* < 0.001).

**Conclusions:**

Studies identified a higher prevalence of mental illnesses among postgraduate students than in the general population. Although students affected are highly educated, their awareness of mental health is not sufficient to know their mental symptoms and seek help. Therefore, we suggest launching wellness programs to enhance their mental health.

**Disclosure of Interest:**

None Declared

